# Stabilization of parameter estimates from multiexponential decay through extension into higher dimensions

**DOI:** 10.1038/s41598-022-08638-7

**Published:** 2022-04-06

**Authors:** Chuan Bi, Kenneth Fishbein, Mustapha Bouhrara, Richard G. Spencer

**Affiliations:** 1 Magnetic Resonance Imaging and Spectroscopy Section, National Institute on Aging, NIH, Baltimore, MD 21224 USA; 2 Magnetic Resonance Physics of Aging and Dementia Unit, National Institute on Aging, NIH, Baltimore, MD 21224 USA

**Keywords:** Mathematics and computing, Physics

## Abstract

Analysis of multiexponential decay has remained a topic of active research for over 200 years. This attests to the widespread importance of this problem and to the profound difficulties in characterizing the underlying monoexponential decays. Here, we demonstrate the fundamental improvement in stability and conditioning of this classic problem through extension to a second dimension; we present statistical analysis, Monte-Carlo simulations, and experimental magnetic resonance relaxometry data to support this remarkable fact. Our results are readily generalizable to higher dimensions and provide a potential means of circumventing conventional limits on multiexponential parameter estimation.

## Introduction

Multiexponential analysis is a longstanding problem in mathematics and physics, with applications in biomedicine^1–3^, engineering^4^, food sciences^5^, the petrochemical industry^6^, and many other settings^7,8^. The goal of many of these analyses, and the problem we will address, is to extract parameter estimates from a real-valued multiexponential decay function of the form

1 $$\begin{aligned} S(t;\{c_i, \tau _i\})=\sum _{i=1}^{n} c_ie^{-t/\tau _i}, \end{aligned}$$

where *n* is the number of underlying monoexponential components, and $\tau _i$ and $c_i$ the decay time constant and amplitude of the *i*-th component. This is a special case of the Laplace transform, itself a special case of the Fredholm equation of the first kind, in which the integrand is the product of an exponential kernel and a sum of delta functions, with all quantities real.

Fitting discrete decay data to Eq. (1), in principle, permits estimation of relative component sizes and decay constants for all components. It is well-known, however, that this process can be severely ill-posed^8,9^; for closely-spaced exponential time constants $\{\tau _i\}$, especially with disparate relative component sizes $\{c_i\}$, there are many sets of distinct decay times and component amplitudes which closely fit the data. A consequence of this is instability in the values of the set of derived parameters in the presence of noise. This can be illustrated through a modification of an example provided by Lanczos^10^.

**Figure 1:**
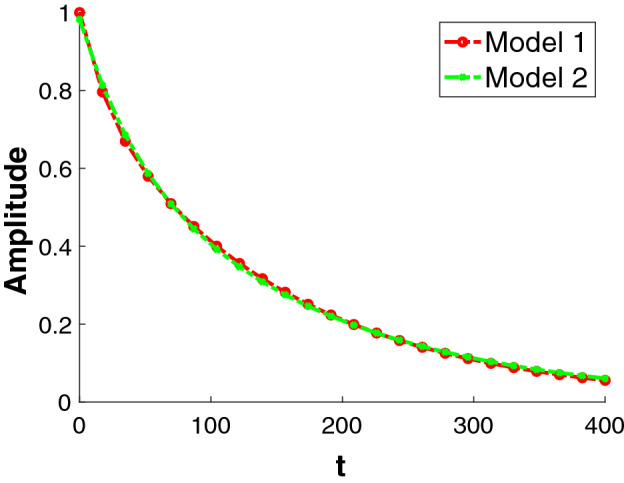
Plot of biexponential decaying signals generated by two different models. The governing equations for the models are $\mathbf {S}_1(\mathbf {t}) = 0.2e^{-\frac{\mathbf {t}}{20}} + 0.8e^{-\frac{\mathbf {t}}{150}}$ and $\mathbf {S}_2(\mathbf {t}) = 0.28 e^{-\frac{\mathbf {t}}{40}} + 0.7 e^{-\frac{\mathbf {t}}{163}}$.

In Fig. 1, we see that there is a near-perfect superposition of two biexponential functions with very different pairs of underlying monoexponentials. Clearly, it is impossible to claim that one of these is more suitable than the other to describe an underlying noisy data set. The ill-posedness of this special case of the inverse Laplace transform (ILT)^9,11–14^ stands in stark contrast to the well-posed Fourier or, equivalently, inverse Fourier, transform.

Many methods^8,15–19^ have been developed for multiexponential analysis, and are effective in particular settings. However, they do not address the fundamental problem of ill-conditioning. In contrast, we show that we can markedly improve the conditioning through two- and higher-dimensional extension of multiexponential decay. We develop this in the context of magnetic resonance relaxometry (MRR), with which, perhaps uniquely among experimental methods, multiexponential data can be generated in one, two, or higher dimensions^2,20–25^. In fact, Celik, et al.^26^ performed a direct experimental confirmation of the increased stability of parameter estimation in 2D MRR, for both nonlinear least squares (NLLS) and non-negative least squares (NNLS) analyses, through a preliminary set of simulations and phantom experiments. However, no analysis was presented to support the empirical results.

The higher-dimensional extensions of MRR are implemented by designing a radio-frequency pulse sequence that is sensitive to two or more intrinsic MR parameters. For example, an experiment that produces data following a 1D biexponential model for transverse decay,

2 $$\begin{aligned} S(TE;c_1, c_2,T_{2,1},T_{2,2})=c_1e^{-TE/T_{2,1}} + c_2e^{-TE/T_{2,2}}, \end{aligned}$$

can be extended into two dimensions by adding sensitization to longitudinal relaxation time:

3 $$\begin{aligned} S(TI,TE;\{c_1,T_{1,1},T_{2,1},c_2,T_{1,2},T_{2,2}\})=c_1(1-2e^{-TI/T_{1,1}})e^{-TE/T_{2,1}} + c_2(1-2e^{-TI/T_{1,2}})e^{-TE/T_{2,2}}, \end{aligned}$$

by incorporating an inversion-recovery pulse sequence module for $T_1$ sensitization followed by a multi-echo acquisition scheme for $T_2$ sensitization, and where the longitudinal relaxation time constants $T_{1,1}$ and $T_{1,2}$ have been introduced. The $T_1$-sensitizing dimension is *indirectly* detected, meaning that the corresponding time variable, *TI*, is a variable time period over which sensitization occurs, rather than a sampling time. This is in contrast to the sampling times *TE* for $T_2$ sensitization. Likewise, diffusion-sensitizing pulses may be incorporated to obtain a signal model very similar to Eq. (3) but with the exponential $T_1$ terms replaced by exponential *ADC* terms, where *ADC* indicates apparent diffusion coefficient. These various 2D experiments^24^ provide more comprehensive chemical characterization of an experimental sample than 1D experiments; here, we are focused on the additional mathematical property of increased stability of the corresponding signal models. These considerations can be generalized to three, and, in principle, higher dimensions. For example, a 2-component signal model incorporating $T_1$, $T_2$, and *ADC* appears as^27,28^:

4 $$\begin{aligned} S(TI, TE, b; \left\{ c_i, T_{1,i}, T_{2,i}, ADC_i\right\} _{i=1}^2) = c_1 e^{-\frac{TE}{T_{2,1}}} \left( 1 - 2e^{-\frac{TI}{T_{1,1}}}\right) e^{-b ADC_1} + c_2 e^{-\frac{TE}{T_{2,2}}} \left( 1 - 2e^{-\frac{TI}{T_{1,2}}}\right) e^{-b ADC_2} \end{aligned}$$

Thus, the purpose of the present work is to extend the preliminary findings of Celik et al.^26^ by providing a statistical theory along with a much more comprehensive set of simulations and experimental data supporting the increased stability of multiexponential analysis in 2D as compared to in 1D. We focus on the archetypical case of the biexponential model, Eqs. (2) and (3). Our main result is that stability improves progressively and markedly for an increasing ratio between the relaxation times, $T_{1,1}$ and $T_{1,2}$, of the two components in the indirect dimension, providing increasing discriminatory information content.

Experimental results are obtained from MRR experiments on a two-component homogeneous gel. For all analyses, we incorporate the consideration that the 2D experiment is of longer duration and provides a greater number of data points than 1D, so that improved stability at a given signal-to-noise ratio (SNR) would be expected. We compensate for this by increasing the signal-to-noise ratio used for 1D Monte-Carlo (MC) simulations and experiments by the square root of the number of indirect dimension measurement points used for the corresponding 2D analysis, $\sqrt{n_{\text {indirect}}}$, as was also done in Celik et al.^26^. We also note that Kim et al.^25^, as part of a comprehensive work on applications, report an improved Cramer-Rao lower bound (CRLB) for the 2D inverse Laplace transform of decaying exponentials in a numerical example; without however exploration of parameter dependencies, and without simulation or experimental results addressing this concept.

For clarity, we note that in contrast to the problem outlined above, multi-dimensional Fourier transform magnetic resonance spectroscopy is a mature field^29^. A fundamental concept in this area is that spectral lines that overlap in a 1D spectrum may be resolved in two- or higher dimensional spectra. However, the Fourier transform is a well-conditioned numerical problem, with condition number of one, so that this is quite distinct from the improvement in condition number from extension of the inverse Laplace transform into higher dimensions which we explore herein. Thus, as a further check on our results, we empirically compare the stability of the 2D to the 1D Fourier transform, finding, as expected, no improvement.

## Theory

In this section, we first work with general linear and nonlinear problems, defined by $\mathbf {G}\mathbf {p}$ and $\mathbf {G}(\mathbf {p})$ respectively. In the mathematical theory for MRR, $\mathbf {G}(\mathbf {p})$ corresponds to the biexponential model in Eq. (3). We will summarize standard results for the variance of parameter estimates determined by linear and nonlinear least squares and show how this formalism can be applied to 2D, and by extension, higher dimensional relaxometry. Linear least squares does not apply directly to parameter estimation from the nonlinear equations Eqs. (2), (3) or (4), but provides the appropriate background for the linearized nonlinear theory to follow.

### Linear theory

An estimate $\mathbf {p}^*$ of a length-*N* parameter vector $\mathbf {p}$ as defined by a linear least squares criterion is:

5 $$\begin{aligned} \mathbf {p}^*=\mathop {\mathrm{argmin}}_{\mathbf {p}}\left\Vert \mathbf {G}\mathbf {p}- \mathbf {d}\right\Vert _2^2 \end{aligned}$$

where the notation indicates that $\mathbf {p}^*$ is the value of $\mathbf {p}$ which minimizes the $l^2$ (Euclidean) norm of the expression within the absolute value. The data vector $\mathbf {d}$ is an *M* dimensional vector of independent Gaussian random variables (RV) of equal variance $\sigma ^2$; the $M \times N$ kernel matrix $\mathbf {G}$ is defined by the physical model of the experiment.

From standard statistical theory and the definition of the pseudoinverse, the covariance matrix of the Gaussian RV, $\mathbf {p^*},$ is an *N*-dimensional Gaussian RV with covariance matrix:

6 $$\begin{aligned} \text {Cov}(\mathbf {p^*})=(\mathbf {G}^T\mathbf {G})^{-1}\sigma ^2 \end{aligned}$$

and

7 $$\begin{aligned} \sigma ^2_{p^*_i}=\left[ (\mathbf {G}^T\mathbf {G})^{-1}\right] _{ii}\sigma ^2. \end{aligned}$$

See the Linear theory section in the Supplemental Information.

### Linearized nonlinear theory

The nonlinear least squares problem corresponding to Eq. (5) is

8 $$\begin{aligned} \mathbf {p}^* = \mathop {\mathrm{argmin}}_{\mathbf {p}} \left\Vert \mathbf {G}(\mathbf {p}) - \mathbf {d}\right\Vert _2^2 \end{aligned}$$

where $\mathbf {G}:\mathbb {R}^N\rightarrow \mathbb {R}^M$. We follow standard methods to derive a result corresponding to Eq. (7)^30^. We start with the first-order approximation of the *j*-th element of $\mathbf {G}(\mathbf {p})$ about a vector $\mathbf {p_0}$:

9 $$\begin{aligned} G_j(\mathbf {p}) \approx G_j(\mathbf {p_0})+\left. \sum _{l}\frac{{\delta }{G_j}}{{\delta }{p_l}}\right| _{\mathbf {p_0}}\cdot {(p_l-p_{0,l})}. \end{aligned}$$

The indicated derivatives are the elements of the Jacobian matrix of $\mathbf {G}$ evaluated at $\mathbf {p_0}$, which we will denote by $\mathbf {B}$ with elements

10 $$\begin{aligned} B_{jl}=\left. \frac{{\delta }{G_j}}{{\delta }p_l}\right| _{\mathbf {p_0}} \end{aligned}$$

Eq. (8) becomes

11 $$\begin{aligned} \mathbf {p}^*=\mathop {\mathrm{argmin}}_{\mathbf {p}}\Vert \mathbf {Bp+(G(p_0)-Bp_0-d)}\Vert _2^2, \end{aligned}$$

which is of the form of Eq. (5), so that

12 $$\begin{aligned} \text {Cov}(\mathbf {p^*})=\left( \mathbf {B}^T\mathbf {B}\right) ^{-1}\mathbf {B}^T \text {Cov}(\mathbf {G(p_0)-Bp_0-d})\left( \left( \mathbf {B}^T\mathbf {B}\right) ^{-1}\mathbf {B}^T\right) ^T. \end{aligned}$$

$\mathbf {G(p_0)}$ and $\mathbf {Bp_0}$ are constants, so $\text {Cov}(\mathbf {G(p_0)-Bp_0-d}) = \text {Cov}(\mathbf {d})$. Then as above,

13 $$\begin{aligned} \text {Cov}(\mathbf {p^*})=\left( \mathbf {B}^T\mathbf {B}\right) ^{-1}{\sigma ^2} \end{aligned}$$

with the diagonal elements again defining the variances of the derived parameters.

This indicates that the condition number for this analysis is defined by $\mathbf {B}$. The complexities of finding a global rather than a local minimum solution to Eq. (8) is a substantial but separate topic; if the linearization is performed about a local minimum, the derived variances will be appropriate for the parameter estimates recovered at that local minimum.

*Cramér-Rao lower bound* (CRLB) theory is an alternative approach to obtaining these results^31–33^; it is also a local analysis. The matrices $\mathbf {G}^T\mathbf {G}$ and ${\mathbf {B}}^T{\mathbf {B}}$ in Eqs. (6) and (13) are *Fisher information matrices*.

## Methods

### One-dimensional analysis

A spin-echo experiment with sampling at echo peaks^1,34^ for a two-component system leads to the signal model of Eq. (2), with a signal vector ${\mathbf {S}}$ defined by $S_j(\mathbf {p})=S(TE_j,{\mathbf {p}})$, and $\mathbf {p}=(c_1,c_2,T_{2,1},T_{2,2})$, with least squares parameter estimation following from:

14 $$\begin{aligned} \mathbf {p}^*=\mathop {\mathrm{argmin}}_{\mathbf {p}}\Vert \mathbf {S}(\mathbf {p}) - \mathbf {d}\Vert _2^2, \end{aligned}$$

where the data vector $$\mathbf {d}$$ is defined according to $$d_j = $$ echo amplitude at time $$TE_j$$. There is no requirement for sampling at equally spaced echo times, though this is conventional and convenient. The *j*-th row of the Jacobian of $$\mathbf {S}$$, which we denote by $$\mathbf {B}$$, corresponds to $$TE_j$$, while column *l* corresponds to the derivatives of $$S_j(\mathbf {p})$$ with respect to the *l*-th element of $$\mathbf {p}$$: $$B_{jl}=\left. \frac{{\delta }{S_j(\mathbf {p})}}{{\delta }p_l}\right| _{\mathbf {p_0}}$$.

A certain degree of prior knowledge of $T_{2,1}$ and $T_{2,2}$ is required to select the vector of measurement times to ensure that the short-time and long-time behavior of the signal is well-sampled; the details of this choice can have a significant effect on fit quality in the presence of noise^35,36^ but is not the subject of the present analysis. The derivatives $B_{jl}$ appearing in the Jacobian are calculated analytically from Eq. (2); for more complicated signal models, they can be computed numerically. The calculation of $\text {Cov}(\mathbf {p^*})$ for different assumed values of $\mathbf {p}$ follows from Eq. (13).

One-dimensional $T_1$ relaxometry experiments may also be implemented; in brief, an initial inversion pulse is followed by a readout pulse after a variable inversion recovery time *TI*. A sequence of $n_{TI}$ inversion recovery times indexed by *k*, $TI_k$, is used to obtain a full data set. The appropriate choice of $\left\{ TI_k\right\}$ is dependent on the values of $T_{1,1}$ and $T_{1,2}$. The corresponding signal model for a two component system is given by the terms in parentheses in Eq. (3), with the $\mathbf {B}$ matrix determined from this. Similar comments apply to one-dimensional diffusion experiments designed to obtain *ADC* values of underlying components.

### Two- and higher-dimensional analysis

There are two independent measurement variables in 2D relaxometry and related experiments^24^. For the version of $T_1-T_2$ experiments we have described, each inversion time $TI_k$ is followed by a number $n_{TE}$ of spin echoes indexed by *j*, denoted by $TE_j$. Individual measurement points are now defined by a pair of times $\left\{ TE_j, TI_k \right\}$, with corresponding data points ${d_{j,k}}$. For two components, this results in a signal described by the two-component model Eq. 3. A full dataset is obtained by stepping through a pre-defined number, $n_{TI}$, of inversion recovery times, acquiring spin-echo data for each.

The least squares minimization problem is defined by the Frobenius norm:

15 $$\begin{aligned} \mathbf {p}^*=\mathop {\mathrm{argmin}}_{\mathbf {p}}\Vert \mathbf {S}\left( \mathbf {p}\right) - \mathbf {d}\Vert _F^2, \end{aligned}$$

a finite double sum over the two-dimensional array of these measurement points; this is obviously independent of the ordering of the measurement time pairs $\{TE_j, TI_k\}$ and their corresponding data points ${d_{j,k}}$. The vector of model parameters is $\mathbf {p}=(c_1,c_2,T_{1,1},T_{1,2},T_{2,1},T_{2,2})$, so that the $\mathbf {B}$ matrix has 6 columns. Each of these columns corresponds to a vectorization of the full 2D grid $\rho$ of measurement points, where $\rho _{j,k}=\{TE_j, TI_k\}$; this is a convenient way to ensure that each column of **B** contains elements corresponding to each measurement point. Having defined $\mathbf {B}$, the calculation of $\text {Cov}(\mathbf {p^*})$ for different underlying parameter sets and measurement points follows as for the 1D case. Experiments comparable to the $T_1-T_2$ experiment, such as $ADC-T_2$ or $ADC-T_1,$ may be analyzed analogously.

Finally, three and higher-dimensional experiments may also be undertaken at the expense of additional acquisition time and more complex data analysis^20^.

### Simulations

For MC simulations, we in general fix all but one of the underlying parameters and show results for a range of this variable parameter. The smaller of the NLLS-derived time constants was assigned the label $T_{2,1}$ and the larger assigned to $T_{2,2}$, with corresponding fractions $c_1$ and $c_2$. In 2D, $T_{1,1}$ and $T_{1,2}$ were similarly assigned to these two components, respectively.

#### One-dimensional simulations

The standard deviations (SD’s) of the derived parameters, $\left( \text {SD}\left( c_1^*\right) ,\text {SD}\left( c_2^*\right) ,\text {SD}\left( T_{2,1}^*\right) , \text {SD}\left( T_{2,2}^*\right) \right)$, for the biexponential model Eq. (2) were plotted as a function of $T_{2,2}$, based on Eqs. (8), (10) and (13). This linear treatment yields results that are simply proportional to the assigned SD of the noise, and hence essentially arbitrarily scaled. We expect a worsening of the condition number of $\mathbf {B}$ and a corresponding increase in the SD of parameter estimates as $T_{2,2}$ approaches $T_{2,1}$.

The linearized treatment outlined above, as well as the equivalent CRLB theory, are local theories and do not directly reflect the global properties of Eq. (8). One potential problem with this is that the evaluation of the Jacobian matrix is taken at the underlying parameter values, which may reflect MC simulations with finite SNR. MC results do not use linearization at the underlying parameter values, but are obtained iteratively from initial guesses near the ground truth, so that while they yield less direct theoretical insight, these results are, in that sense, considerably more general. These simulations are again displayed as a function of $T_{2,2}$. The MC simulations were performed by adding $N_{\text {noise}}$ noise realizations of Gaussian noise to each decay curve for a given set of parameters and performing an NLLS fit for each. SNR was defined as the ratio of the maximum signal amplitude to the noise SD. The mean and SD’s of recovered parameters were then calculated over the set of noise realizations. Quantitative agreement between the analytic linearized solution and the MC results is not expected due to the linearization used to derive Eq. (9), and the evaluation of the Jacobian matrix $\mathbf {B}$ at the true underlying model parameters rather than at the parameters recovered by NLLS, and dependence on the details of the numerical NLLS algorithm. This effect can be minimized through use of very high SNR values in directly comparing the MC with the analytic results; we have selected SNR=10,000 which, in fact, is close to our experimental SNR (see below).

For both the analytic linearized covariance analysis and the MC analyses, we assumed evenly-spaced echo times $\left\{ TE_j\right\}$ ranging from 8 ms to 512 ms in 8 ms increments; the number of echo times was therefore $n_{TE}=64$. For all MC simulations, 1000 noise realizations were used. The initial guesses used for NLLS were random numbers within a specified range of the underlying parameter values. Parameter estimation was implemented using the MATLAB function lsqcurvefit , an unconstrained Levenberg-Marquardt algorithm.

#### Two-dimensional simulations

For the 2D simulations defined by Eq. (3), additional parameters $\left( T_{1,1}, T_{1,2} \right)$ are introduced and need to be determined. The analytical and MC simulations were designed analogously to the 1D versions described above. Results are presented by fixing $T_{1,1}=1000$ ms and varying $T_{1,2}$. The parameter covariance matrix and the condition number of the Jacobian matrix for the linearized analytical calculations was determined from Eqs. (10) and (13). For MC simulations, the full set of 6 estimated parameters was again determined for $N_{\text {noise}} = 1000$ realizations of noise-corrupted data for a given set of model parameters. The set of $n_{TI} = 25$ inversion recovery times $\left\{ TI_k \right\}$ ranged from 50 ms to 4850 ms in increments of 200 ms. The echo times $\left\{ TE_j\right\}$ were the same as for the 1D simulations. The total number of measurements was $n_{TI} \times n_{TE} = 1600$. SNR was defined as for the 1D case.

#### 1D versus 2D simulations

The 2D experiment collects an $n_{TI}$-fold greater number of data points than 1D, leading to increased acquisition time by approximately the same factor. In practice, an equal-time comparison is of greatest interest; an equal-time 1D experiment would exhibit SNR which is a factor $\sim \sqrt{n_{TI}}$ greater than the corresponding 2D experiment. In other words, given the same experimental time, one can perform either a single 2D acquisition or $n_{TI}$ 1D acquisitions, and we seek to compare the stability of these two approaches. This has been incorporated into all of our MC simulations and experimental analyses, as was also done in Celik et al.^26^.

Otherwise, our comparisons of 1D and 2D experiments used the same set of echo times $\left\{ TE_j \right\}$ and underlying common parameter values. For the 2D simulations, the effect of the difference between or ratio of $T_{1,1}$ and $T_{1,2}$ on the precision of parameter estimation was of greatest interest.

In contrast to the ILT, the inverse Fourier transform (FT) is analytically well-posed and, in the discrete form, well-conditioned^9^. It therefore serves as a type of negative control on our results for the ILT, to demonstrate the fact that the results we obtain in the latter case are due to improvement in conditioning rather than simply to expanding dimensionality. See SI (Supplementary Information), Fig. 5 for details of this analysis.

#### Three-dimensional simulations

The extension to higher dimensions is straightforward. For example, Eq. (4) represents a signal model incorporating $T_1$, $T_2$ and diffusion effects. Each of the two components is now characterized by a triplet $\{T_{1,i}, T_{2,i}, ADC_i\}$, with *i* indexing the two components. The number of experimental data points is $n_{TE}\times n_{TI} \times n_b$, where $n_b$ is the number of discrete diffusion-sensitizing measurements. The linearization of this problem, following the formalism above for linearization of 1D and 2D models, is straightforward, including the construction of the Jacobian and the covariance matrix.

### Experimental methods

One-dimensional $T_2$ and 2D $T_1 - T_2$ experiments were performed on a 5% agarose gel consisting of two cylindrical plugs doped respectively with 0.05% and 0.15% w/v CuSO$_4$. Each component was weighed to estimate expected relative signal fractions. To facilitate shimming, the plugs were immersed in perfluorocarbon liquid (3M Fluorinert FC-770, Sigma-Aldrich, St. Louis, MO) and positioned between two home-built polyetherimide (Ultem) plastic plugs. The plugs were separated by a 1mm thick poly(tetrafluoroethylene) spacer to prevent diffusion of copper ions between them. After insertion into a 10mm NMR tube, the two-component gel was placed in a 10mm transmit-receive SAW resonator (m2m Imaging, Australia) within the magnet and scanned using an Avance III 400MHz widebore microimaging spectrometer (Bruker Biospin, Rheinstetten, Germany). Sample temperature was maintained at 4.0 ±0.1 $^{\circ }$C using cold air from a vortex tube (Exair, Cincinnati, OH).

Non-localized spectroscopic data were acquired using a CPMG multi-spin echo sequence consisting of rectangular RF pulses of duration 20 $\upmu$s (90$^{\circ }$) and 40 $\upmu$s (180$^{\circ }$), yielding 2048 echo peak intensities at $TE = 0.4, 0.8, \cdots , 819.2$ ms for each spin excitation, followed by a recovery delay of 2s. For two-dimensional $T_1-T_2$ experiments, each CPMG pulse train was preceded by a 40 $\upmu$s rectangular inversion pulse and an inversion recovery delay *TI*, which was incremented nonlinearly from 15ms to 2s in 24 steps. 1D $T_2$ experiments, without the inversion recovery preparation module, were performed with 30 averages to yield similar total scan time to that of the 2D experiments. Each 1D or 2D experiment was repeated 100 times to produce data with different noise realizations. Total scan time for each experiment was 1 hour 38 minutes.

Spin-echo imaging experiments were performed to measure $T_1$ and $T_2$ relaxation times in each gel independently. In each experiment, a 3 mm axial slice was positioned through a single gel, with in-plane field-of-view 10 mm $\times$ 10 mm and matrix size 64 $\times$ 64. Excitation and refocusing were performed using 1ms hermite 90$^{\circ }$ and bandwidth-matched hermite 180$^{\circ }$ pulses, respectively. All imaging experiments were performed without signal averaging.

Monoexponential $T_2$’s were measured using a CPMG sequence in which the read pre-phase gradient directly preceded the readout gradient to minimize diffusion effects. Sequence parameters included: acquisition bandwidth $= 81.5$ kHz, interpulse delay $TR = 2$ s, and $TE = 4.7, 9.4, \cdots , 601.6$ ms. Mean magnitude intensities for all gel pixels were fit to a three-parameter exponential decay function $A + M_0*\exp (-TE/T_2)$ in Bruker ParaVision 5.1 software; the offset term was incorporated to account roughly for the Rician noise floor.

Monoexponential $T_1$ values were measured similarly, using a progressive saturation experiment in which *TR* was varied nonlinearly from 15 ms to 3 s in 8 steps. Acquisition bandwidth $= 50$ kHz and $TE = 6.0$ ms were employed. Data were fit to the function $A + M_0*(1-\exp (-TR/T_1))$.

The relaxometry experiments were performed in spectroscopic mode, that is, with no spatial-encoding gradients. The acquired data was therefore of the form of a single complex value for each acquisition time. Each data point was phased individually along the real axis to maintain full amplitude without any magnitude operation, as is standard experimental practice in MR relaxometry. Thus, the noise in our experiments was Gaussian, as required for the strict validity of Eqs. (6) and (12).

## Results

### One-dimensional analyses

#### Analytic calculation

**Figure 2:**
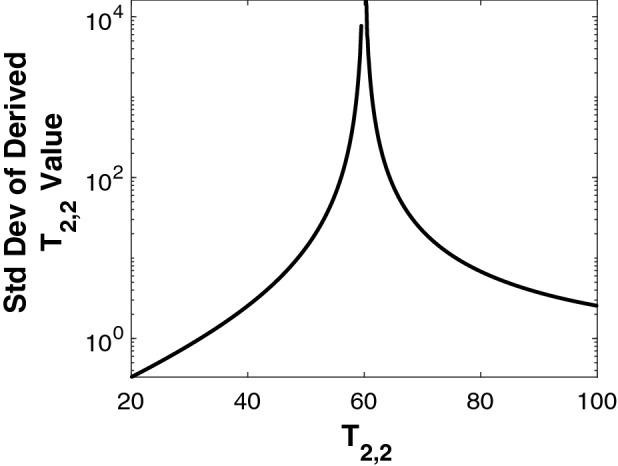
Analytical calculation of the standard deviations for $T_{2,2}$ recovery from the biexponential 1D model, as a function of $T_{2,2}$. $T_{2,1} = 60$ ms throughout. Values are obtained from the square root of the diagonal elements of the covariance matrix defined by Eq. (13).

Fig. 2 shows the linearized results for the standard deviation of $T_{2,2}$ derived from the model described by Eq. (2), as a function of $T_{2,2}$, with $c_{1}= 0.3$, $c_{2}=0.7$ and $T_{2,1}=60$ ms. SNR was set to 800, though this value appears only as a multiplicative constant and does not otherwise enter the calculation.

We see that the variance increases greatly, and asymptotically approaches infinity, as $T_{2,2}$ approaches $T_{2,1}$. Similar results are seen for the SD of all other derived parameters (See Figure 1 in the SI). Correspondingly, the condition number of the Jacobian matrix $\mathbf {B}$ approaches infinity as $T_{2,2}$ approaches $T_{2,1}$, as shown in Fig. 2 of the SI. In fact it may readily be confirmed by inspection that $\mathbf {B}$ is singular in this limit; $\mathbf {B}^T\mathbf {B}$ is then also singular, so that the right-hand side of Eq. (13) is undefined.

This limit represents the coalescence of the two exponential terms in Eq. 2. The time constant of the resulting monoexponential expression follows easily from NLLS analysis, but of course it is impossible to separate the two underlying components. We also see that $\text {cond}(\mathbf {B})$ becomes very large even before this limit is attained, so that the calculation of $\text {Cov}(\mathbf {p^*})$ becomes effectively meaningless and is therefore excluded from Fig. 2.

The theoretical calculations in Eq. (13) in effect assume infinite SNR through the fact that the Jacobian is always calculated at the correct underlying values, with finite SNR incorporated into the formalism only through multiplication by the noise variance.

#### Monte-Carlo simulations

**Figure 3:**
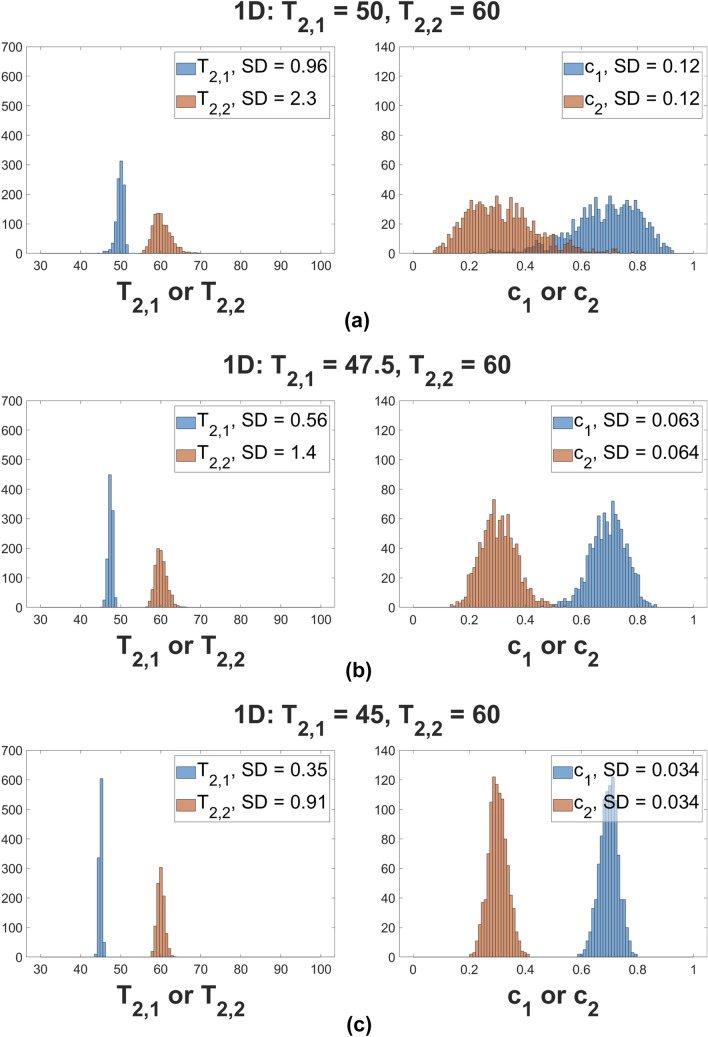
Histograms of one-dimensional MC simulation results for parameter recovery from the 1D biexponential model Eq. 2. All rows share the same underlying values $\left( c_1, c_2\right) = \left( 0.7, 0.3\right)$. Underlying $T_2$ values are as indicated. The standard deviations, denoted SD, are calculated from the distributions obtained with MC simulations. For all MC simulations, the smaller value of the derived time constants is assigned to $T_{2,1}$ and the larger to $T_{2,2}$, with their fractions $c_1$ and $c_2$ assigned correspondingly.

Fig. 3 shows MC results indicating the improvement in stability as $T_2$ values become increasingly different. Results are shown for SNR= 10000 over 1000 noise realizations.

**Figure 4:**
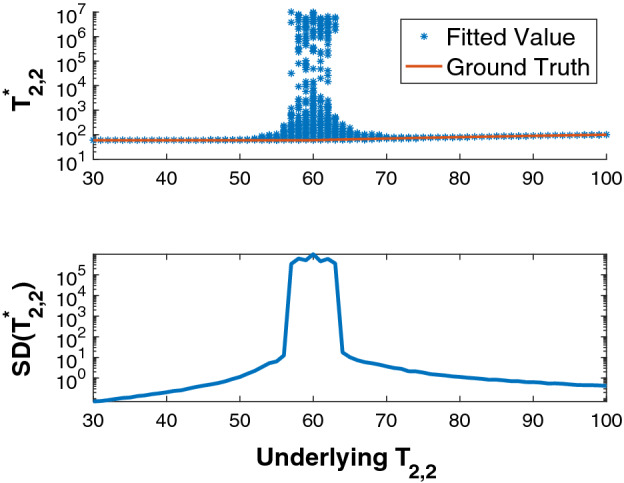
The upper panel shows a scatterplot of the recovered values, $T_{2,2}^*$, of $T_{2,2}$ over 1000 noise realizations for each value of underlying $T_{2,2}$, with the remaining parameters fixed at $\left( c_1,c_2,T_{2,1} \right) = \left( 0.3, 0.7, 60\text { ms}\right)$. The smaller value of the derived time constants is assigned to $T_{2,1}$, and the larger to $T_{2,2}$. SNR = 10000. In the upper panel, correct underlying values of $T_{2,2}$ are indicated with the red line, with corresponding values of the recovered $T_{2,2}$ shown as asterisks. As $T_{2,2}$ increasingly differs from $T_{2,1}$, accuracy and precision both improve greatly. The SD’s for the recovered values of $T_{2,2}$ are shown in the lower panel and are seen to be largest in the regime $T_{2,2} \approx T_{2,1}$, and to decrease as these values progressively differ.

These results can be extended by plotting histograms of recovered parameter values for a range $T_{2,2}$ values. Fig. 4 shows this for recovered $T_{2,2}$ values over 1000 noise realizations with $\text {SNR} = 10000$; high SNR was selected in order to minimize the potential effect of local minima, so that the MC results could be more directly compared to the linearized treatment. Parameter SD’s were calculated for each value of $T_{2,2}$. Note that SD indicates the standard deviation of the distribution obtained from MC simulations and should be distinguished from the $\sigma$ defining the standard deviation of a Gaussian distribution. As seen, accuracy increases and the SD of estimates decreases as the ratio of $T_{2,1}$ to $T_{2,2}$ increasingly differs from unity, in agreement with Fig. 3. Results for the other parameters are similar.

In this extremely high SNR case, the pattern of the calculated SD’s is very similar to that found from the linearized theory. This is to be expected, since the latter in effect assumes infinite SNR in that the Jacobian is always calculated at the correct underlying values. In contrast, MC results are independent of linearization and are not reliant on a high-SNR approximation. The MC results we show in the remainder of this paper, for more realistic SNR, are expected to show the same trends as in the linearized theory, but not to agree in detail. In particular, for moderate SNR, we expect large parameter SD across a much larger range of the independent variable as compared to the linearized result. Nevertheless, for both linearized analytic and MC calculations, we expect maximal SD in the regime $T_{2,1} \approx T_{2,2}$. A more exact correspondence between these methods is not to be expected.

### Two-dimensional analyses

#### Analytic calculation

**Figure 5:**
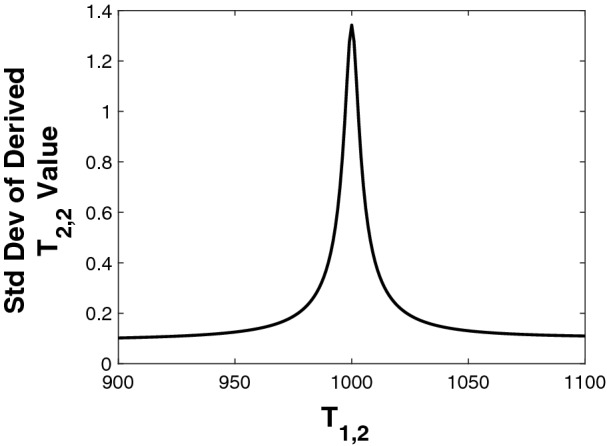
Results of analytical linearized calculation of SD for recovery of biexponential 2D model parameter $T_{2,2}$ as a function of $T_{1,2}$, with other parameters fixed. Values are obtained from the square root of the diagonal elements of the covariance matrix defined by Eq. (13).

Fig. 5 shows results based on Eq. (13) for the recovery of $T_{2,2}$ from 2D $T_1-T_2$ experiments. The SD’s of the derived values are shown as a function of $T_{1,2}$, varying from 800 ms to 1200 ms with other parameters fixed at the values $\left( c_1, c_2, T_{2,1},T_{2,2}, T_{1,1}\right) = \left( 0.3, 0.7, 60\text { ms}, 45\text { ms}, 1000\text { ms}\right)$. Similar results are obtained for the SD’s of all other parameters (See SI, Fig. 3). Additive Gaussian noise was again assumed and entered only as an overall multiplicative constant.

As expected, the SD for each parameter attains its maximum for $T_{1,2} = T_{1,1} = 1000$ ms, and decreases as $T_{1,2}$ deviates from this value. This result is the major finding of this work, indicating the statistical basis for our previous empirical results^26^. In particular, this supports the stabilization of parameter estimation for the biexponential model through introduction of a second dimension with disparate values for $T_1$’s.

We expect that the behavior of the SD should correspond to the condition number of the Jacobian matrix $\mathbf {B}$, and therefore of $\mathbf {B}^T \mathbf {B}$ for the linearized problem, as described in the theory section.

**Figure 6:**
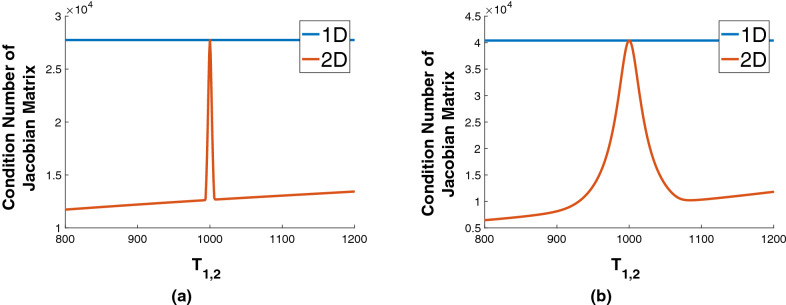
Condition number of the Jacobian matrices for 1D and 2D models. Left: Results for $\mathbf {p}^* = \left( c_1,c_2 , T_{2,1},T_{2,2}\right) = \left( 0.3,0.7,60\text { ms},45\text { ms} \right)$, with $T_{1,1}=1000$ ms for the 2D case and calculations performed across a range of $T_{1,2}$. Right: Analogous results for $\mathbf {p}^* = \left( c_1,c_2,T_{2,1},T_{2,2} \right) = \left( 0.8,0.2, 90\text { ms},160\text { ms} \right)$. As seen, the condition number for the 2D case in which $T_{1,1}=T_{1,2}$ is close to that of the 1D case.

Fig. 6 shows that the condition number of the Jacobian matrix for 2D experiments with differing $T_{1,1}$ and $T_{1,2}$ is smaller than for corresponding 1D experiments. When $T_{1,1}=T_{1,2}$, the two condition numbers are approximately equal. This indicates that the stability of parameter estimation from 2D experiments is greater than for 1D except in the regime of this special case. The equivalence of the 1D experiment to the 2D experiment for equal $T_1$’s can only be approximate, since the condition number of the latter has a dependence on the set of $T_1$-sensitizing *TI* values; this variable does not exist in 1D. Fig. 8 in the SI is a threedimensional view corresponding to Fig. 5 above.

In comparing the results of Fig. 5 with Fig. 2, and from Fig. 6, we see that the $T_{1,1} = T_{1,2}$ behavior in 2D mimics the $T_{2,1}=T_{2,2}$ behavior in 1D. Stability improves in 1D as the ratio $T_{2,1}/T_{2,2}$ departs from unity, while it improves in 2D as the ratio $T_{1,1}/T_{1,2}$ similarly departs from unity. However, even with $T_{11}=T_{12}$, the condition number will remain finite as long as $T_{21} \ne T_{22}$.

#### Monte-Carlo simulation results

**Figure 7:**
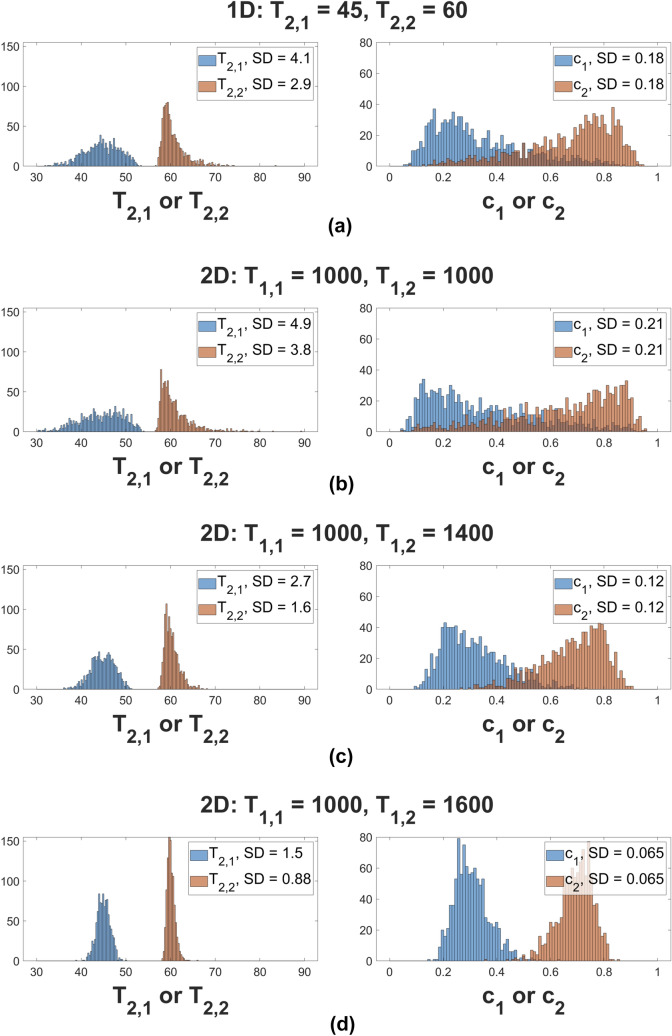
Histograms of 1D and 2D MC simulations over 1000 noise realizations. Row 7a shows 1D results with underlying parameters as indicated. Panels 7b, 7c, and 7d show 2D results; all plots share the same underlying values with $\left( c_1, c_2, T_{2,1}, T_{2,2}\right) = \left( 0.3, 0.7, 45 \text { ms}, 60 \text { ms}\right)$, and with pairs of $T_1$ values as indicated for the 2D simulation.

Fig. 7 compares MC results for the stability of the 1D and 2D biexponential analyses. Parameter recovery was performed over 1000 noise realizations. Underlying parameter values were $c_1 = 0.3$, $c_2 = 0.7$, $T_{2,1} = 45$ ms, and $T_{2,2} = 60$ ms, with, in addition, $T_{1,1} = 1000$ ms. Results are shown for three values of $T_{1,2}$. SNR was set to $\text {SNR}_{2D} = 400$ for the 2D analysis, and $\text {SNR}_{1D} = 400\times \sqrt{n_{TI}} = 2000$ in 1D. The histograms show recovered values of the indicated parameters.

As seen, the histograms for the 1D analysis and for the 2D analysis with equal $T_1$’s are essentially indistinguishable. Precision increases in 2D as the $T_1$’s progressively differ, demonstrating the potential for improved stability of 2D $T_1-T_2$ experiments as compared to 1D, even on the equal-time basis in which the SNR of the 1D experiment is, in this case, $\sqrt{n_{TI}}$=5-fold greater than that of the 2D experiment.

**Figure 8:**
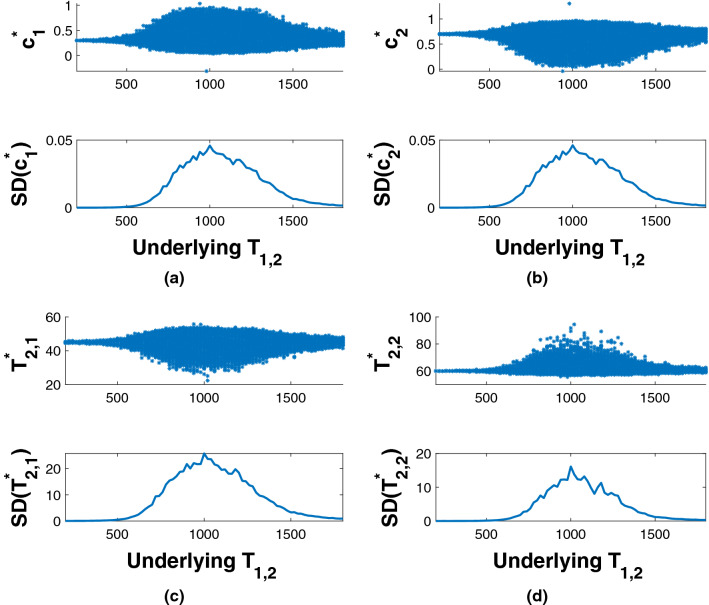
The uppermost and third rows show recovered values of the indicated parameter over 1000 noise realizations, with underlying parameter values fixed at $\left( c_1,c_2,T_{2,1},T_{2,2},T_{1,1} \right) = \left( 0.3, 0.7, 45\text { ms}, 60\text { ms}, 1000\text { ms}\right)$, and with $T_{1,2}$ varying across the indicated range. The SD for each recovered parameter as a function of $T_{1,2}$ is shown beneath its histogram. The SD’s are largest in the regime $T_{1,2} \approx T_{1,1}$, and decrease as these values progressively differ.

The results of Fig. 7 can be extended as shown in Fig. 8. Variation in NLLS parameter estimation is shown for 2D analysis as a function of $T_{1,2}$. Recovered values are correctly obtained for $T_{1,2}$ increasingly different from $T_{1,1}$. The SD’s were calculated over 1000 noise realizations for each parameter set. As seen, the SD of the estimates decrease as the ratio of $T_{1,2}$ to $T_{1,1}$ deviates from unity, in agreement with the results shown in Fig. 3 in the SI. In addition, the stability of the 2D experiment depends on the ratio of the $T_1$’s rather than on their absolute separation; see Fig. 4 in the SI. A three-dimensional version of Fig. 8, showing a MC calculation of SD as a function of $T_{1,1}$ and $T_{2,1}$, is provided in Fig. 9 in the SI.

### Three-dimensional analyses

**Figure 9:**
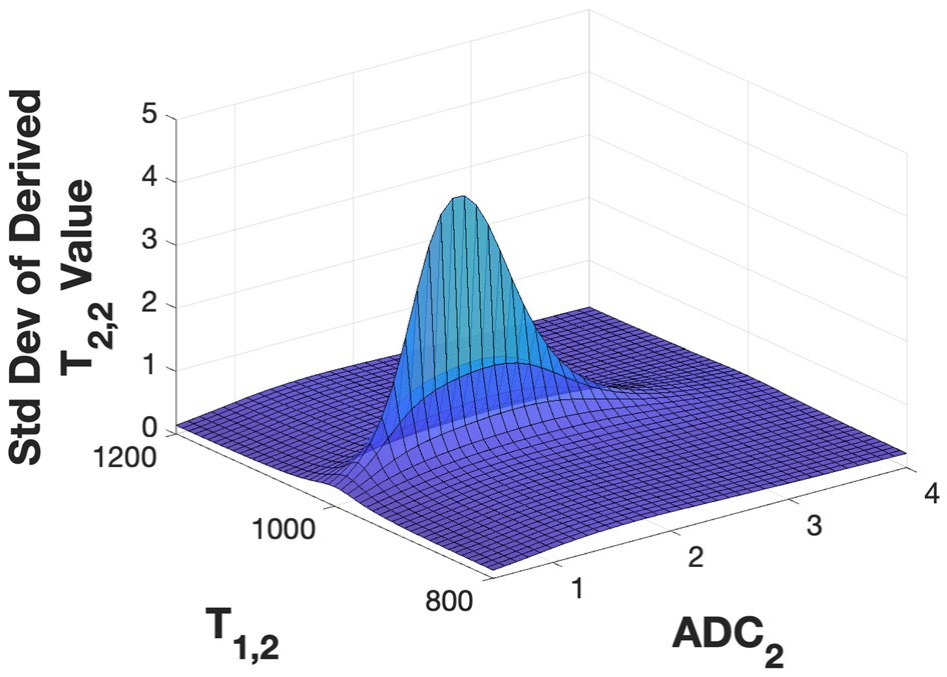
Results of linearized analytical calculation of the SD of recovered values for $T_{2,2}$ in the biexponential 3D model, as a function of indirect dimension parameters $\left( T_{1,2}, ADC_2\right)$, with other parameters fixed at $c_1 = 0.3$, $c_2 = 0.7$, $T_{2,1} = 45\text { ms}$, $T_{2,2} = 60\text { ms}$, $T_{1,1} = 1000\text { ms}$, and $ADC_1= 1.5$ mm$^2$/ms. The SD values are obtained from the square root of the corresponding diagonal elements of the covariance matrix defined by Eq. (13).

We provide a more condensed treatment of the further extension of the ILT of multiexponential decay to three dimensions (3D). As described in the three-dimensional simulations section, expressions for the SD of parameter estimates can be derived through linearization analogously to the 2D case. We illustrate this for the $T_1-T_2-ADC$ signal model, with fixed values of $\left( c_1,c_2,T_{2,1},T_{2,2},T_{1,1},ADC_1 \right) = \left( 0.7, 0.3, 45 \text { ms}, 60\text { ms}, 1000\text { ms}, 1.5 \text { mm}^2/\text {ms}\right)$, and varying $T_{1,2}$ and $ADC_2$. We used nine evenly-spaced diffusion sensitizing values $\left\{ b_l \right\}$, ranging from 0 ms/mm$^2$ to 2 ms/mm$^2$ in increments of 0.25 ms/mm$^2$. The dimensions of *b* are inverse to those of *ADC*. The values of $\left\{ TE_j\right\}$ and $\left\{ TI_k\right\}$ were taken to be the same as in the one and two-dimensional simulations sections. The number of measurement points is $n_{TE} \times n_{TI} \times n_b = 14,400$. However, we reiterate that typically, the 64 values of *TE* that provide $T_2$-sensitization are acquired at no additional time cost through a multi-echo acquisition, so that the duration of the experiment is largely proportional to $n_{TI} \times n_b$. The standard deviation of $T_{2,2}$ is illustrated in Fig. 9 as a function of the indirect dimension values of $T_{1,2}$ and $ADC_2$. Similar results are seen for the SD of all other derived parameters; see Fig. 6 in the SI.

**Figure 10:**
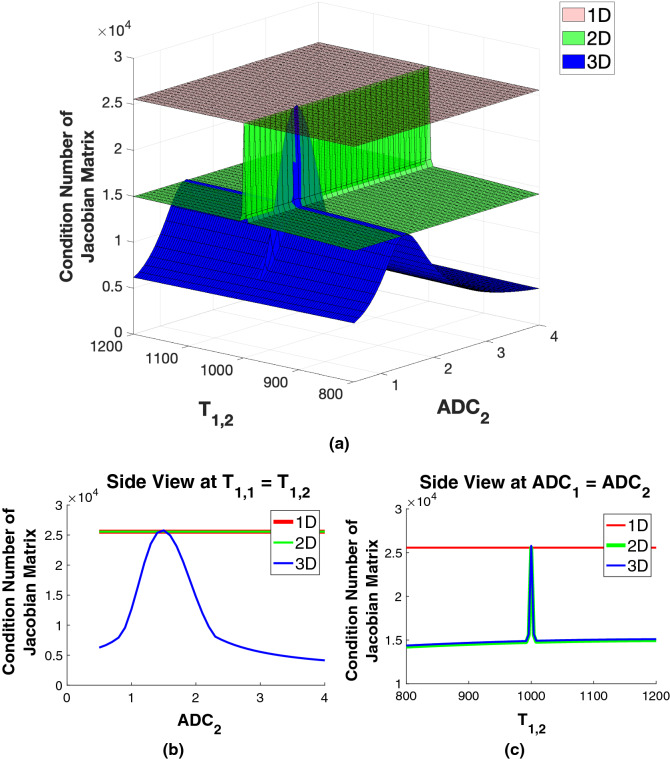
Top: Condition numbers for the Jacobian matrix for 1D, 2D, and 3D models evaluated over a range of $T_{1,2}$ and $ADC_2$ values. Bottom left: Perspective view of the slice through $ADC_1=ADC_2$ for the case $T_{1,1} = T_{1,2}$. Bottom right: Perspective view of the slice through $ADC_1 = ADC_2$.

Figure 9 shows that the maximum SD for $T_{2,2}$ estimation, that is, greatest degree of instability, occurs for $T_{1,2} = T_{1,1}$ and $ADC_2 = ADC_1$. This is exactly analogous to the previous results in 1D and 2D. The basis for this can be seen from calculating the condition number of the Jacobian matrix. This is shown in Fig. 10, calculated using the same set of parameters as in Fig. 9.

The uppermost plot in Fig.10 shows that the condition numbers are largest in 1D and for the 2D and 3D analyses when the indirect dimension parameter values are equal. The condition number is then seen to decrease when the indirect dimension parameter values become disparate. Thus, for equal indirect dimension parameter values, no additional information is provided by that dimension, and the condition numbers become essentially equal to those for the next-lower dimension. The lower left plot shows the rough equality of condition numbers for all three dimensions when the indirect dimension parameter values are equal, with the condition number decreasing as $ADC_2$ becomes increasingly different from $ADC_1$. The lower right plot shows the marked increase in condition number as $T_{1,2}$ approaches $T_{1,1}$, and the decrease as these two values become increasingly disparate. For 1D, the condition number is plotted as a constant across the $T_{1,2}$ and $ADC_2$ axes, while the condition number for the two-dimensional problem is plotted as constant along the $ADC_2$ axis. These results indicate that the condition number for the 1D model is effectively an upper bound for the 2D model, which is itself an approximate upper bound for the 3D model.

### Experimental results

**Figure 11:**
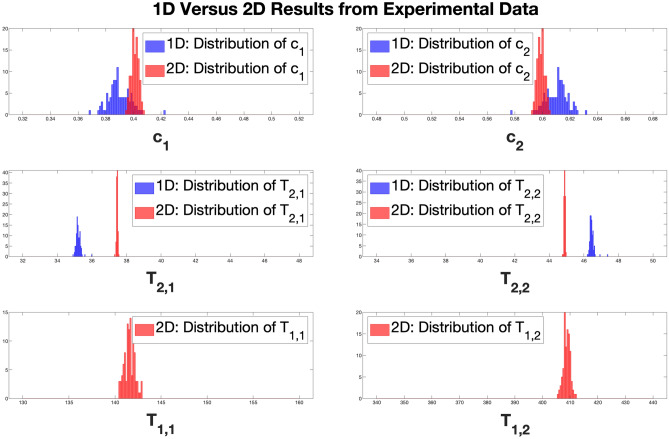
Histograms of the fitted values for the indicated parameters derived using NLLS from 100 sets of experimental data. The comparisons in the upper two rows are for 1D versus 2D. Note that the mirror image appearance of $c_1$ and $c_2$ arises from the constraint that $c_1 + c_2 = 1$.

The results of the gel experiments are shown in Fig. 11. We note that the estimated $T_1$ and $T_2$ values from the spin-echo imaging experiments are $\left( c_1, c_2\right) = (0.47, 0.53)$, $\left( T_{2,1}, T_{2,2}\right) = 36.3 \text { ms}, 45.9 \text { ms}$, and $\left( T_{1,1}, T_{1,2}\right) = \left( 157 \text { ms}, 405 \text { ms}\right)$. SNR of the experimental data is $\sim 9000$ for the 1D experiment and $\sim 2000$ for the 2D.

Histograms displaying the fit values for $c_1$ and $c_2$ over 100 experimental data sets, each with an independent realization of experimental noise, are shown in the upper two panels; results for the estimates of $T_{2,1}$ and $T_{2,2}$ are shown in the middle panels, and histograms of estimated $T_{1,1}$ and $T_{1,2}$ values are shown in the bottom two panels.

The 1D NLLS analysis of the signal obtained from the double gel sample yields $\text {SD}\left( c_1^*\right) \approx 8\times 10^{-3}$, $\text {SD}\left( c_2^*\right) \approx 8\times 10^{-3}$, $\text {SD}\left( T_{2,1}^*\right) \approx 1.37\times 10^{-1}$ ms, and $\text {SD}\left( T_{2,2}^*\right) \approx 1.34\times 10^{-1}$ ms; the 2D $T_1 - T_2$ NLLS analysis with $\frac{T_{1,2}}{T_{1,1}} \approx 2.9$ yields substantially smaller standard deviations for all four parameters, with $\text {SD}\left( c_1^*\right) \approx 2.7\times 10^{-3}$, $\text {SD}\left( c_2^*\right) \approx 3.7\times 10^{-3}$, $\text {SD}\left( T_{2,1}^*\right) \approx 3.4\times 10^{-2}$ ms, and $\text {SD}\left( T_{2,2}^*\right) \approx 3.5\times 10^{-2}$ ms. Additional simulations confirm that the stability of the 2D reconstruction shows substantial improvement as compared to the 1D results, where the same TI, TE and SNR values as in the experiment are used. See Fig. 7 in the SI for more details.

Quite separately from stability issues, we note that there are unmodeled effects in these experimental data that likely contribute to the differences in derived mean values between the two methods, although such bias is not the topic of the current work. Among these effects are the non-i.i.d. Gaussian noise encountered in actual experimental practice, along with the fact that the effect of noise on the bias in 1D and 2D NLLS is a complicated function of noise characteristics and SNR. In addition, the underlying $T_2$ values of the two components of the gel sample will not be delta-function monoexponentials, but rather distributions, albeit narrow. An analysis of the bias in noisy 2D NLLS would represent a significant undertaking beyond the scope of the present paper.

## Discussion

Parameter estimation for multi-component exponential decay has been studied for over 200 years, dating back at least to Prony^15^, and has remained an active area of research through the present day^7,8,18,37^. Many algebraic and numerical approaches for this have been established and reviewed^8,18^, including Prony’s^15,38,39^ and the Laplace-Padé methods^40^, several implementations of nonlinear least squares analysis (itself a topic of longstanding study, likely originating with Gauss^41^ )^42–44^, and others^7,8,18,45^. More recently, machine learning methods have been applied to this problem^19,46^.

Bromage^38^ made important comments regarding the conditioning of the 1D biexponential model as a function of the separation of the *c* and the $T_2$ values. In the one-dimensional analyses section of the present work, we present comparable results, but also provide condition numbers derived from a linearized analytic treatment, and parameter variances for both the MC and analytic approaches. Varah^14^ analyzed the uniqueness and stability of the biexponential 1D problem in the context of both discrete (NLLS) and continuous ($L^2$ norm of the misfit) analyses. Least squares surfaces as defined by the objective function in Eq. (14) were presented for a range of parameters to illustrate the ill-conditioning of the biexponential problem. Shrager^17^ provided an extensive perspective on the difficulties of multiexponential model fitting to experimental data, especially in the context of ill-conditioning. An alternative Bayesian approach to deriving parameter uncertainties in this setting has also been presented^16,47^ in terms of relaxation rates $\frac{1}{T_2}$. Nevertheless, in spite of the range of techniques presented in the literature, the fundamental difficulty in deriving the amplitudes and time constants for multiexponential, and even for biexponential, decay remains; it is intrinsic to the redundancy in the family of exponential functions, with the possibility that very different exponential models may provide nearly identical values^8–10^. Especially in the presence of unavoidable experimental noise, parameter extraction in such cases is an intractable problem. In practical terms, this means that derived parameters are extremely unstable with respect to noise^48,49^.

Previous analyses have been developed within established fundamental limitations^50^. However, perhaps uniquely among experimental sciences, magnetic resonance studies permit the experimental implementation of two- and higher-dimensional experiments yielding data exhibiting multiexponential decay. Evaluation of the increased stability of these models is the central idea of the present work. This is a fundamental departure from the vast body of previous work on 1D biexponential or multiexponential analysis. Celik et al.^26^ provided an initial report of this improved conditioning; however, they presented no underlying theory, and minimal simulation and experimental results. In the present manuscript, we provide a statistical foundation for this result, as well as a much more extensive theoretical and numerical analysis, along with substantially expanded experimental results. Overall, our results provide a potential means of circumventing conventional limits on multiexponential parameter estimation.

Although the approximate analytic and MC results presented here are fully supportive of each other, there are some obvious differences. First and foremost, the MC calculation is exact in the sense that it involves no explicit assumptions. In contrast, the analytic approach is a linearization. In addition, the results of the MC simulations depend to a certain extent on initial guesses and details of the implementation of the nonlinear optimization. We used a commercial, highly-developed, code for this, but these dependencies remain. Further, the computations involved in the approximate analytic analysis become less reliable as the condition number of the $\mathbf {B}$ matrix increases. Likewise, the calculation of variances is based on a first order Taylor expansion around the true parameter values, so that the minimizer $\mathbf {p}^*$ must be near the defined underlying parameter values, that is, there is an implicit assumption of limited bias. Similarly, the Jacobian matrix in the linearized treatment is always evaluated at the correct underlying parameters, which does not realistically correspond to the MC results. Nevertheless, both approaches, linearized theoretical modelling and MC simulations, reflect the main result of this paper, which is the increased stability of parameter estimation for the ILT of biexponential decay in 2D with distinct indirect dimension parameters.

A significant issue is that the condition number of the Jacobian matrix in the linearization of Eq. (8) is unit-dependent. This follows from the fact that the parameters to be estimated, component sizes and relaxation times, are of different dimensions. There appears to be no fully satisfactory resolution of this issue other than to choose reasonable and conventional units that are internally consistent within the given analysis.

Extensions of the present work would include dimensional considerations for stability as a function of discretization^50,51^, and within the framework of NNLS, for which the stabilizing effect of increased dimensionality was also demonstrated empirically by Celik et al.^26^. Such further analyses would be of particular interest given the recent advances in accelerating data acquisition for higher dimensional MRR^6,28,52,53^.

In conclusion, we have demonstrated a fundamental improvement in the stability of biexponential decay analysis through extension into higher dimensions.

## Supplementary Information

Supplementary Information.

## Data Availability

All datasets generated and analysed are available in the following Zendo repository https://doi.org/10.5281/zenodo.4558564.
