# Supplementary material for: Stabilization of parameter estimates from multiexponential decay through extension into higher dimensions
Source: Sci Rep. 2022 Apr 6;12:5773. doi: 10.1038/s41598-022-08638-7 (PMC8986819; doi:10.1038/s41598-022-08638-7)
Supplement: Supplementary file 1 — Supplementary Information. [file 41598_2022_8638_MOESM1_ESM.pdf]

Supplementary Information: Stabilization of parameter estimates from multiexponential decay through extension into higher dimensions

Chuan Bi,*[1], Kenneth Fishbein[1], Mustapha Bouhrara[2], and Richard G. Spencer[1]

[1]Magnetic Resonance Imaging and Spectroscopy Section, National Institute on Aging, NIH, Baltimore, MD, USA 21224
[2]Magnetic Resonance Physics of Aging and Dementia Unit, National Institute on Aging, NIH, Baltimore, MD, USA 21224
[*]chuan.bi@mail.nih.gov

ABSTRACT

Analysis of multiexponential decay has remained a topic of active research for over 200 years. This attests to the widespread importance of this problem and to the profound difficulties in characterizing the underlying monoexponential decays. Here, we demonstrate the fundamental improvement in stability and conditioning of this classic problem through extension to a second dimension; we present statistical analysis, Monte Carlo simulations, and experimental magnetic resonance relaxometry data to support this remarkable fact. Our results are readily generalizable to higher dimensions and provide a potential means of circumventing conventional limits on multiexponential parameter estimation.

Linear theory

An estimate $\mathbf{p}^*$ of a length-N parameter vector $\mathbf{p}$ as defined by a linear least squares criterion is:

$$\mathbf{p}^* = \underset{\mathbf{p}}{\operatorname{argmin}} \|\mathbf{G}\mathbf{p} - \mathbf{d}\|_2^2 \tag{1}$$

where the notation indicates that $\mathbf{p}^*$ is the value of $\mathbf{p}$ which minimizes the l^2 (Euclidean) norm of the expression within the absolute value. The data vector $\mathbf{d}$ is a column vector of length M; the $M \times N$ kernel matrix $\mathbf{G}$ is defined by the physical model of the experiment. The elements of the vector $\mathbf{Gp}$ are the estimated values of the data generated by the model for parameter set $\mathbf{p}$, so that $(\mathbf{Gp} - \mathbf{d})$ is the vector of residuals. The columns of $\mathbf{G}$ correspond to model parameters, while the rows correspond to measurement times or other variables defining the set of measurements. We will consider only the case in which $M > N$, and $rank(\mathbf{G}) = N$.

The Moore-Penrose pseudoinverse solution to Eq. (1) is[1]:

$$\mathbf{p}^* = \left(\mathbf{G}^T\mathbf{G}\right)^{-1}\mathbf{G}^T\mathbf{d} \tag{2}$$

This solution is usually not implemented directly due to the potentially large condition number of $\mathbf{G^TG}$, but is very useful for theoretical considerations, such as in the following.

The covariance between two random variables (RV's) p_i and p_j is defined as

$$\text{Cov}(p_i, p_j) = \mathbb{E}[(p_i - \mathbb{E}[p_i])(p_j - \mathbb{E}[p_j])], \tag{3}$$

where $\mathbb{E}$ denotes statistical expectation value, and describes the linear relationship between p_i and p_j. Cov($\mathbf{p}$) is defined by

$$[\text{Cov}(\mathbf{p})]_{i,j} = \text{Cov}(p_i, p_j) \tag{4}$$

The diagonal elements are the variances of the corresponding RV's: $[\text{Cov}(\mathbf{p})]_{i,i} = \sigma_{p_i}^2$. From standard statistical theory the covariance matrix of a linear transformation of a Gaussian RV $\mathbf{p}$, $\mathbf{Gp}$, is itself a Gaussian RV with covariance matrix:

$$\text{Cov}(\mathbf{Gp}) = \mathbf{G}\,\text{Cov}(\mathbf{p})\,\mathbf{G^T}. \tag{5}$$

By Eq. (2), $\mathbf{p}^*$ is an N-dimensional Gaussian RV, so that its covariance matrix, from Eq. (5), is

$$\mathrm{Cov}(\mathbf{p}^*) = \left(\mathbf{G}^T\mathbf{G}\right)^{-1}\mathbf{G}^T\mathrm{Cov}(\mathbf{d})\left(\left(\mathbf{G}^T\mathbf{G}\right)^{-1}\mathbf{G}^T\right)^T \tag{6}$$

In the usual case of uncorrelated data points with equal variance σ^2, $\mathrm{Cov}(\mathbf{d}) = \mathbf{I}_M\sigma^2$, where $\mathbf{I}_M$ is the M-dimensional identity matrix, so that

$$\mathrm{Cov}(\mathbf{p}^*) = (\mathbf{G}^T\mathbf{G})^{-1}\sigma^2 \tag{7}$$

and

$$\sigma^2_{p_i^*} = \left[(\mathbf{G}^T\mathbf{G})^{-1}\right]_{ii}\sigma^2. \tag{8}$$

One-dimensional analyses

Fig. 1 shows the linearized results for the standard deviation of parameters derived from the biexponential model as a function of $T_{2,2}$, with $c_1 = 0.3$, $c_2 = 0.7$ and $T_{2,1} = 60$ ms. SNR was set to 800, though this value appears only as a multiplicative constant and does not otherwise enter the calculation.

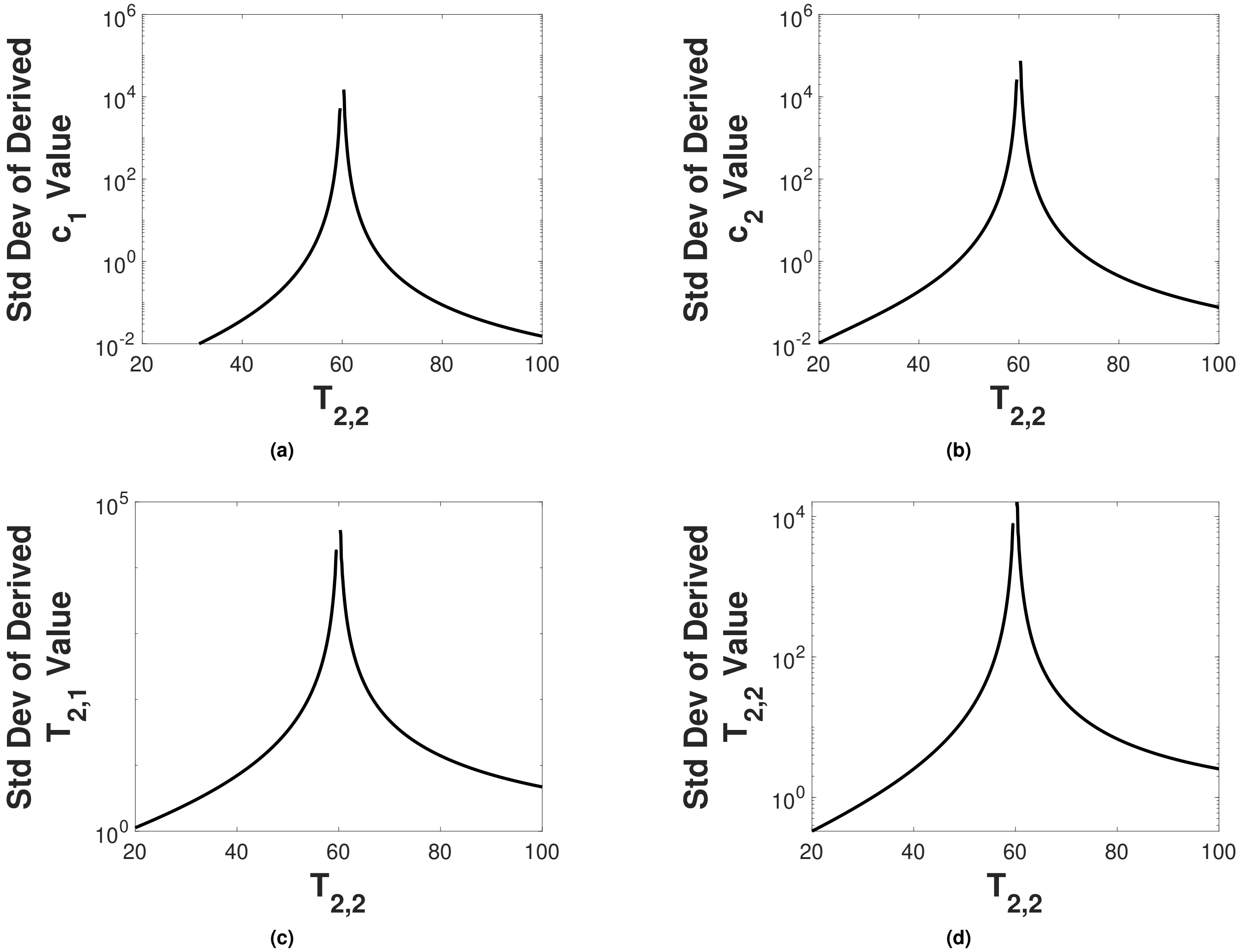

Figure 1. Analytical calculation of the standard deviations for recovery of biexponential 1D model parameters as a function of $T_{2,2}$. $T_{2,1} = 60$ ms throughout. Values are obtained from the square root of the diagonal elements of the covariance matrix defined by Eq. (13) in the main manuscript.

Two-dimensional analyses

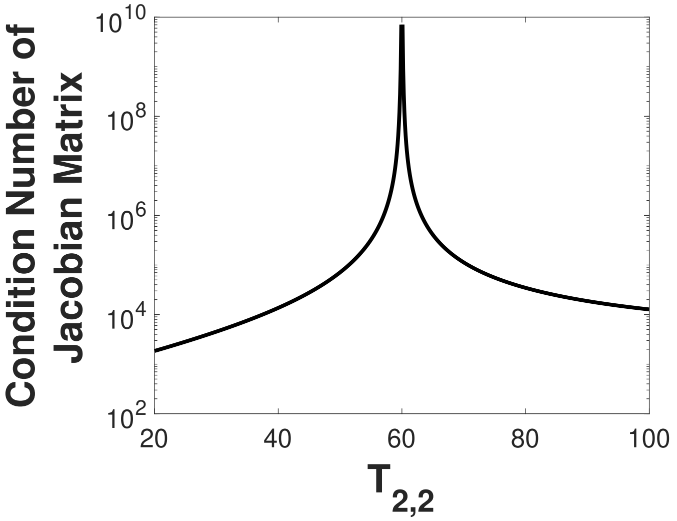

Figure 2. Plot of condition number of the Jacobian matrix **B** as a function of $T_{2,2}$ for the 1D biexponential model Eq. (2) in the main text, with matrix elements defined according to Eq. (10) in the main manuscript.

Comparison of the 1D and 2D inverse Fourier transform

In contrast to the ILT, the inverse Fourier transform (FT) is analytically well-posed and, in the discrete form, well-conditioned[2]. It therefore serves as a type of negative control on our results for the ILT, to demonstrate the fact that the results we obtain in the latter case are due to improvement in conditioning rather than simply to expanding dimensionality.

With frequencies in the FT formulation taking the place of the relaxation time constants in the Laplace transform formulation, the two-component 1D model is:

$$S(t;c_1,c_2,\nu_1,\nu_2) = c_1 \exp\left(2\pi \mathrm{i}\, \nu_1 t\right) + c_2 \exp\left(2\pi \mathrm{i}\, \nu_2 t\right) + \varepsilon \tag{9}$$

where c_1 and c_2 are component sizes, ν_1 and ν_2 the corresponding frequencies, and ε additive Gaussian noise in both real and complex channels. The inverse problem is the determination of the four parameters $\mathbf{p}^* = (c_1^*, c_2^*, \nu_1^*, \nu_2^*)$ from noisy data **d**; we use the MATLAB function `fft` for this.

The corresponding 2D model is:

$$S(t,\tau;c_1,c_2,\nu_1,\nu_2,\mu_1,\mu_2) = c_1 \exp\left(2\pi \mathrm{i}\left(\nu_1 t + \mu_1 \tau\right)\right) + c_2 \exp\left(2\pi \mathrm{i}\left(\nu_2 t + \mu_2 \tau\right)\right) \tag{10}$$

where the μ_i are the frequencies in the second dimension, with time variable τ. The six parameters $\mathbf{p}^* = (c_1^*, c_2^*, \nu_1^*, \nu_2^*, \mu_1^*, \mu_2^*)$ need to be determined from 2D noisy data **d** via the 2D FT; we implemented this using the MATLAB function `fft2`. To demonstrate that the increased stability of the ILT for biexponential decay in 2D is a consequence of improvement in condition number rather than simply arising from the introduction of an additional dimension, we performed similar analysis for the FT. This is a well-conditioned orthogonal transform with a condition number of one for the discrete FT matrix[2]. We further note that the FT and inverse FT (IFT) are identical except for the sign in the exponential kernel, which has no effect on condition number. Fig. 5 shows histograms for recovery of the parameters from the 1D IFT model, Eq. (9), and the 2D IFT model, Eq. (10). As for the ILT results above, we compared 1D and 2D experiments on an equal-time basis, so that the SNR in 1D was greater than in 2D.

Figure 5 compares the 1D and 2D IFT. The details of the results depend on SNR and the fact that the 1D model has greater SNR than the 2D model. Nevertheless, it is clear that the stability of the FT does not improve through the introduction of an additional dimension and with an increased difference between the model parameters in the second dimension.

Three-dimensional analyses

See Fig. 6 for the SD for all derived parameters.

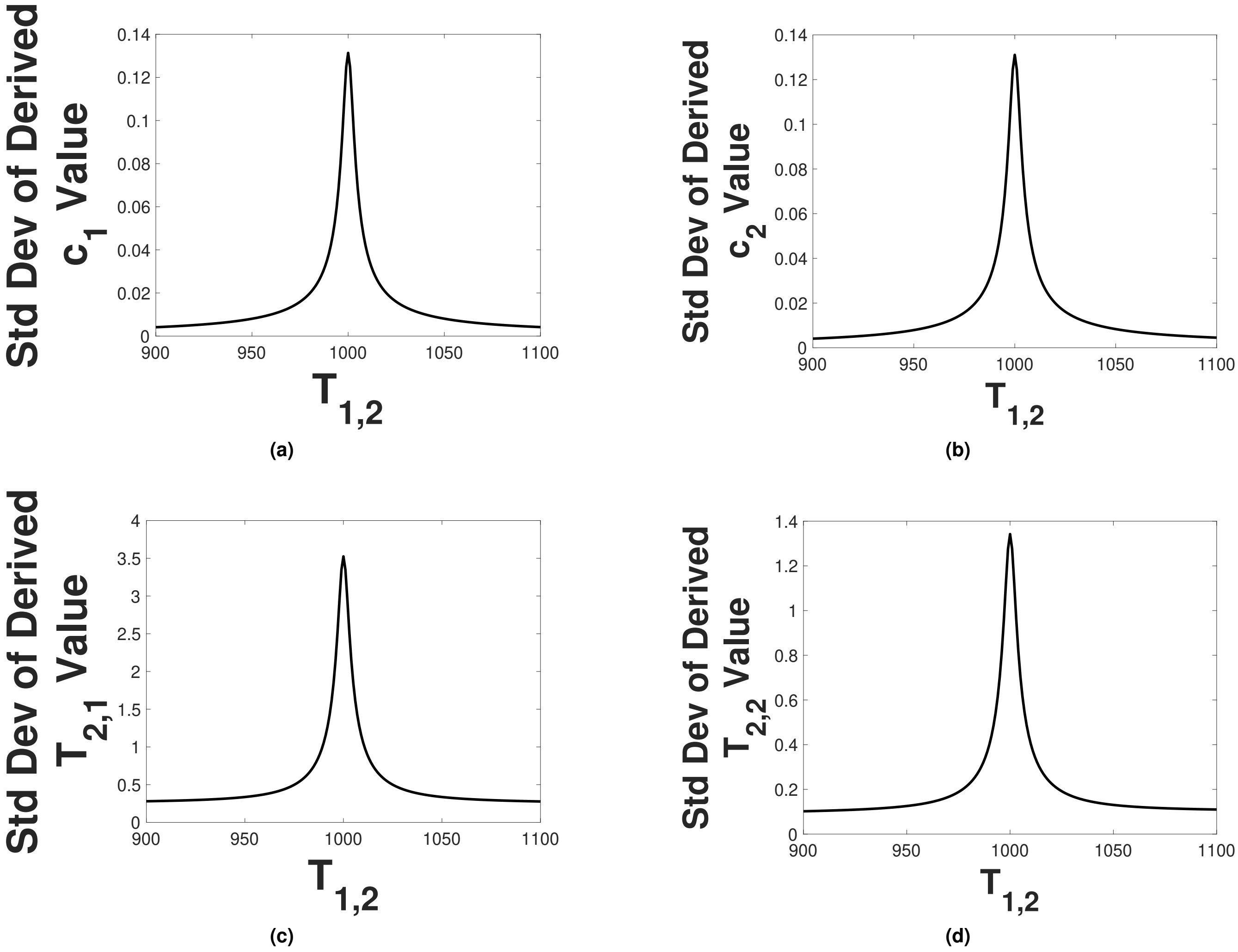

Figure 3. Results of analytical linearized calculation of SD's for recovery of biexponential 2D model parameters as a function of $T_{1,2}$, with other parameters fixed. Values are obtained from the square root of the diagonal elements of the covariance matrix defined by Eq. (13) in the main manuscript. Panels 3a - 3d show results for $\left(\sigma_{c_1}, \sigma_{c_2}, \sigma_{T_{2,1}}, \sigma_{T_{2,2}}\right)$, respectively.

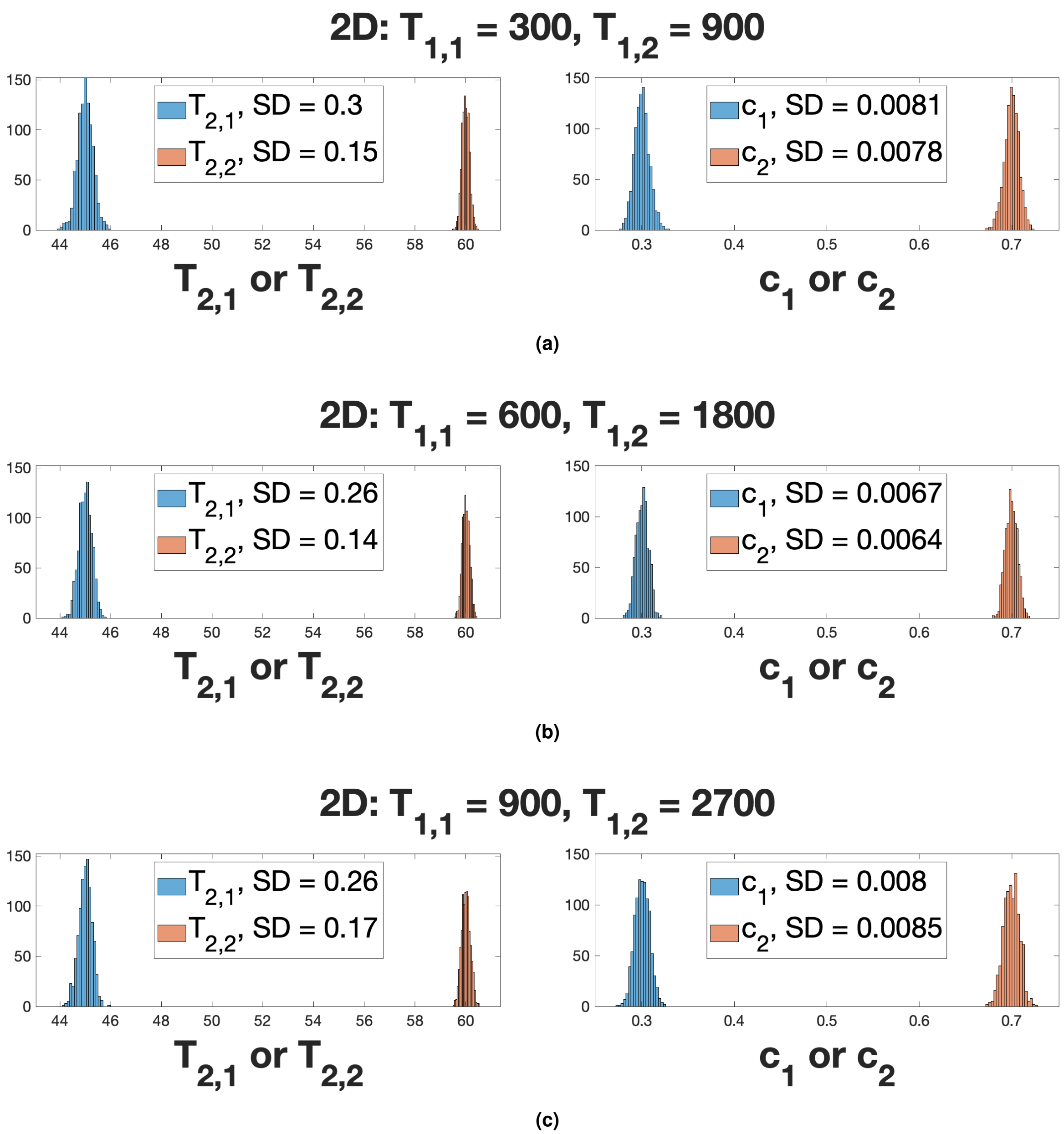

Figure 4. Histograms of 1D and 2D Monte-Carlo simulation results, indicating the constancy of parameter estimation stability for given $T_{1,1}/T_{1,2}$. All rows share the same underlying values with $(c_1, c_2, T_{2,1}, T_{2,2}) = (0.3, 0.7, 45\text{ ms}, 60\text{ ms})$.

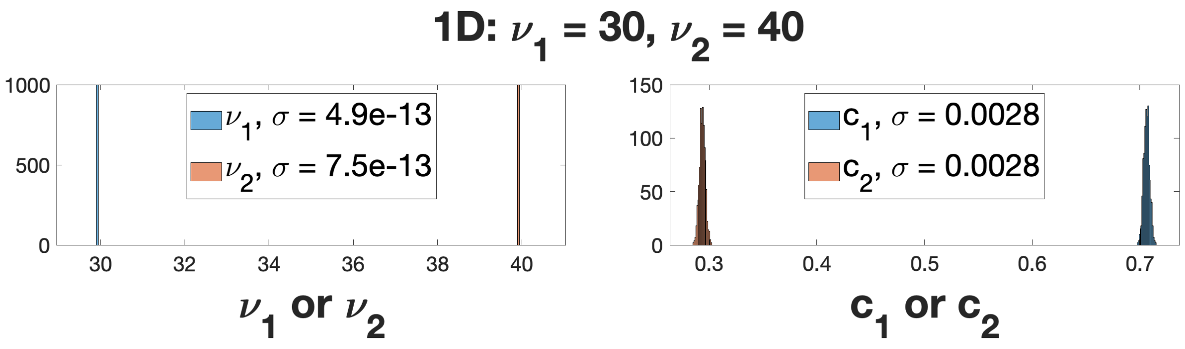

(a)

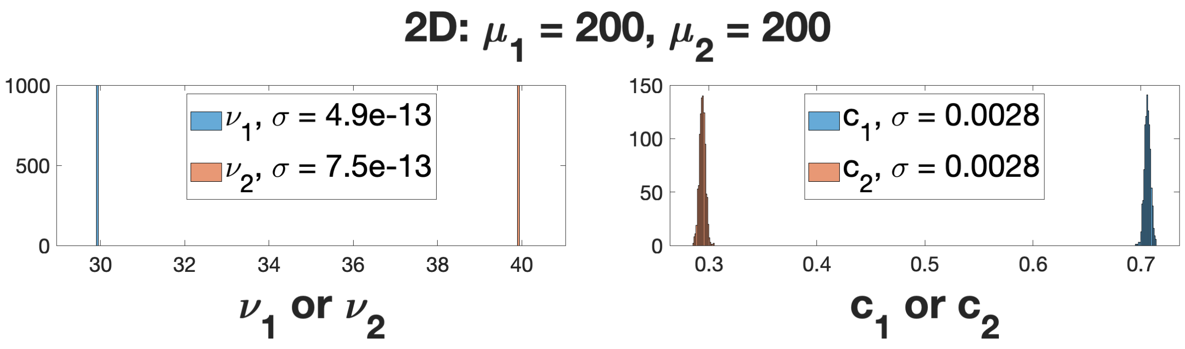

(b)

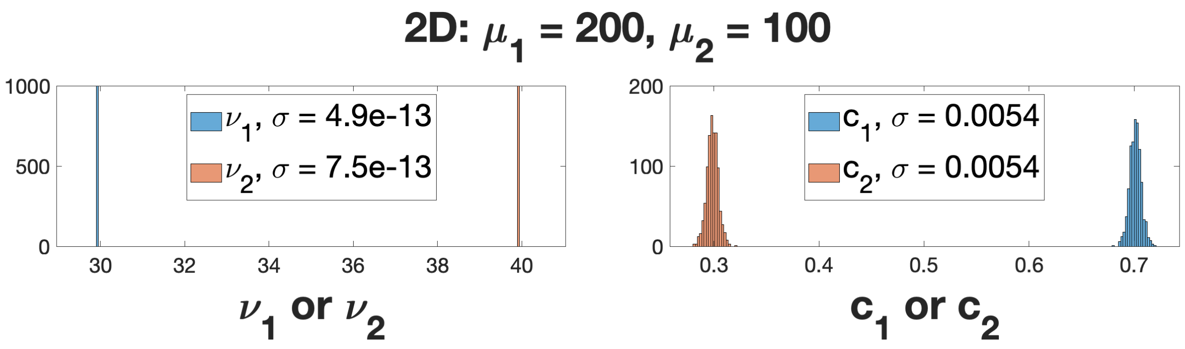

(c)

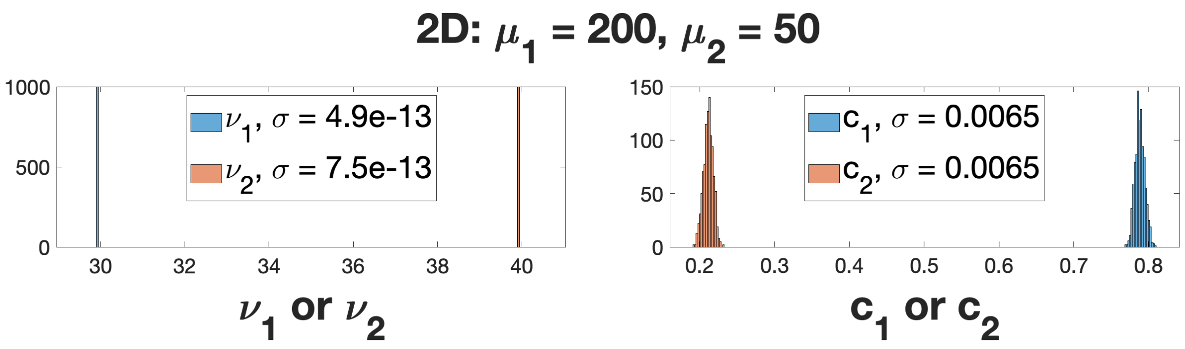

(d)

Figure 5. Histograms of 1D and 2D IFT Monte-Carlo simulation results. The upper two rows show histograms of recovered parameters for the 1D (top row) and 2D models shown in Eqs. (9) and (10). The underlying model parameters are $(c_1, c_2, \nu_1, \nu_2) = (0.7, 0.3, 30 \text{ ms}, 40\text{ms})$, with the addition, for the 2D model, of $\mu_1 = \mu_2 = 200$ ms. The lower three rows show results for 2D models with increasing disparity between the values of μ_1 and μ_2.

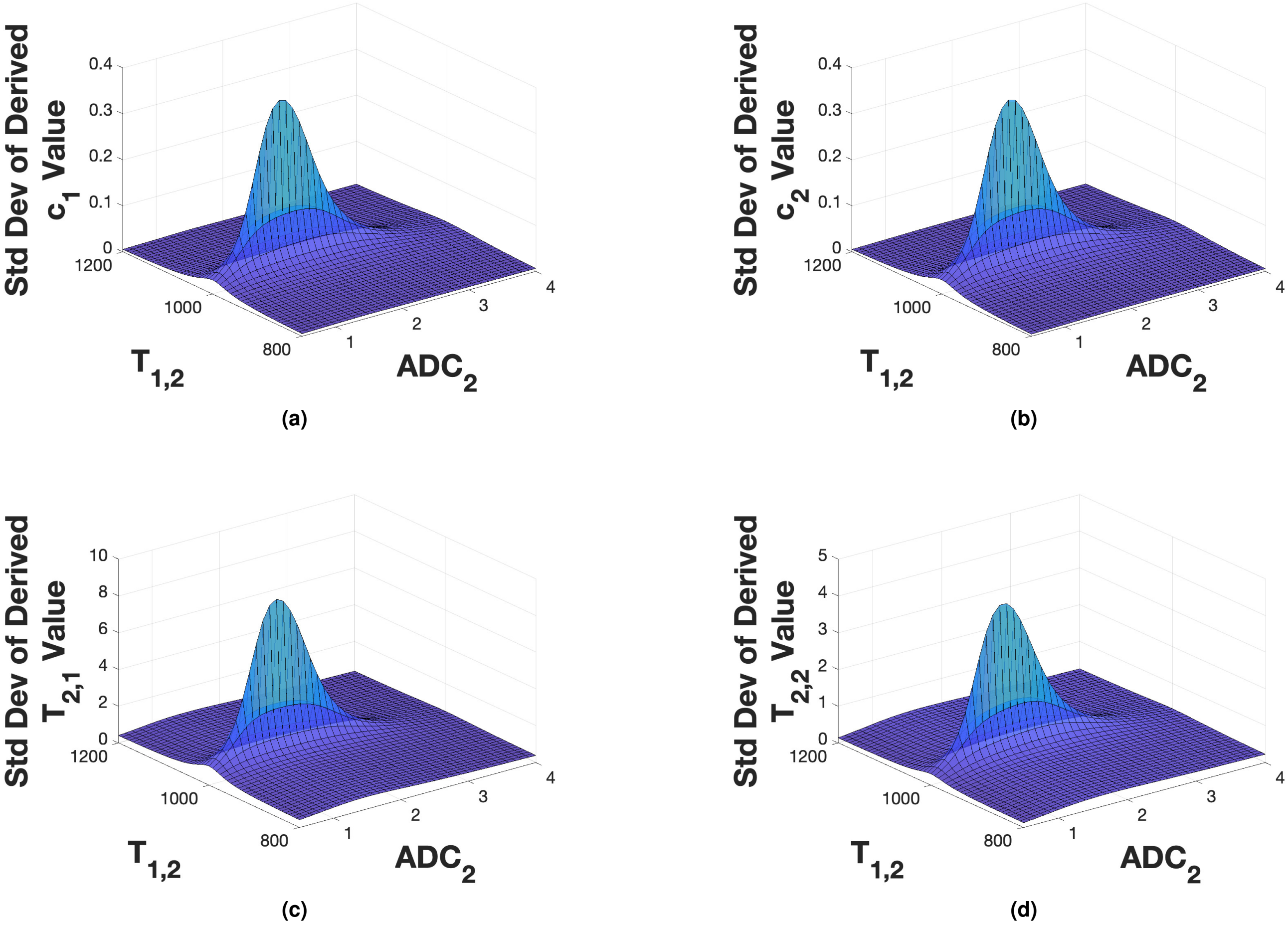

Figure 6. Results of linearized analytical calculation of parameter SD's for a biexponential 3D model as a function of indirect dimension parameters $(T_{1,2}, ADC_2)$, with other parameters fixed at $c_1 = 0.3$, $c_2 = 0.7$, $T_{2,1} = 45$ ms, $T_{2,2} = 60$ ms, $T_{1,1} = 1000$ ms, and $ADC_1 = 1.5$ mm^2/ms. The SD values are obtained from the square root of the corresponding diagonal elements of the covariance matrix defined by Eq. (13) in the main text. Panels 6a - 6d indicate the results for $\left(\sigma_{c_1}, \sigma_{c_2}, \sigma_{T_{2,1}}, \sigma_{T_{2,2}}\right)$, respectively.

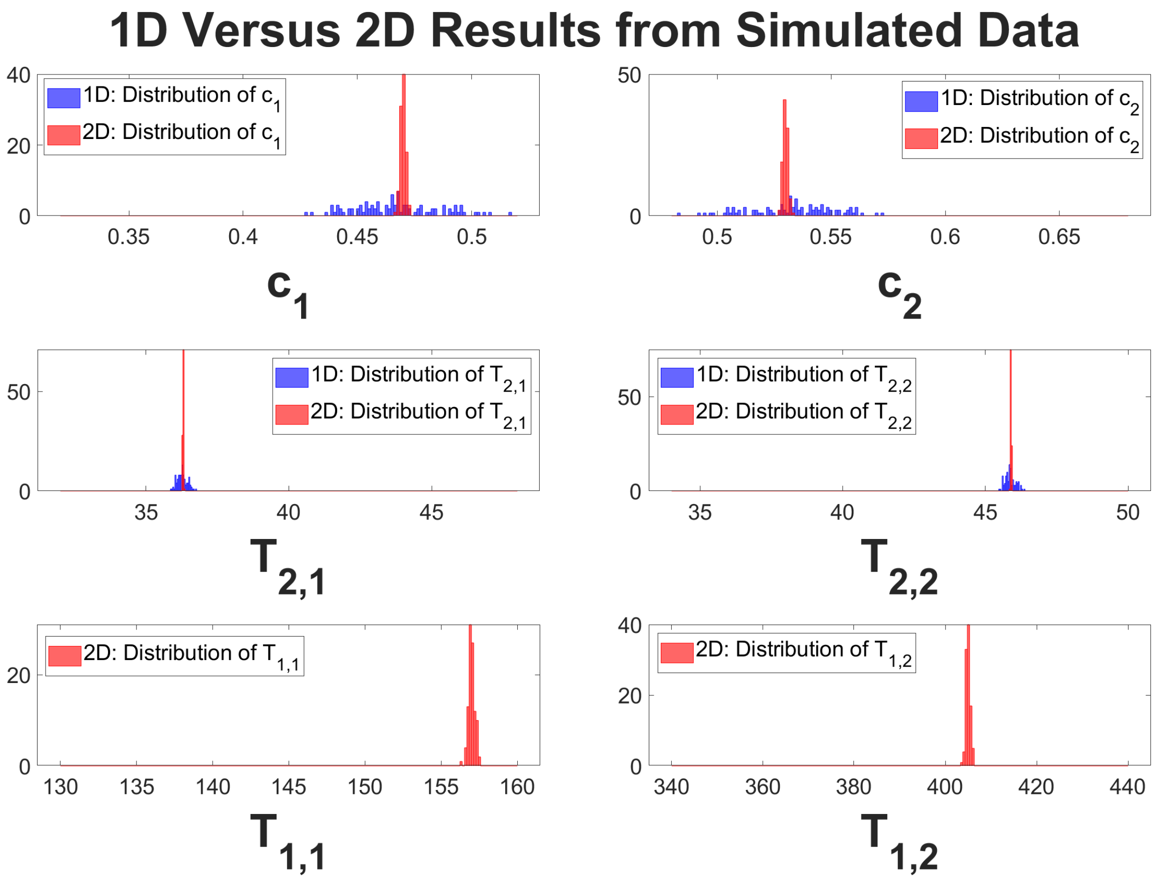

Figure 7. Histograms of the fitted values for the indicated parameters derived using NLLS from 100 sets of simulated data. Linearly spaced TE values are used, where $TE = 0.4, 0.8, \cdots, 819.2$ ms, yielding 2048 echoes; for the inversion recovery delay TI, which was incremented non-linearly from 1 5ms to 2 s in 24 steps. The comparisons in the upper two rows are for 1D versus 2D. Note that the mirror image appearance of c_1 and c_2 arises from the constraint that $c_1 + c_2 = 1$.

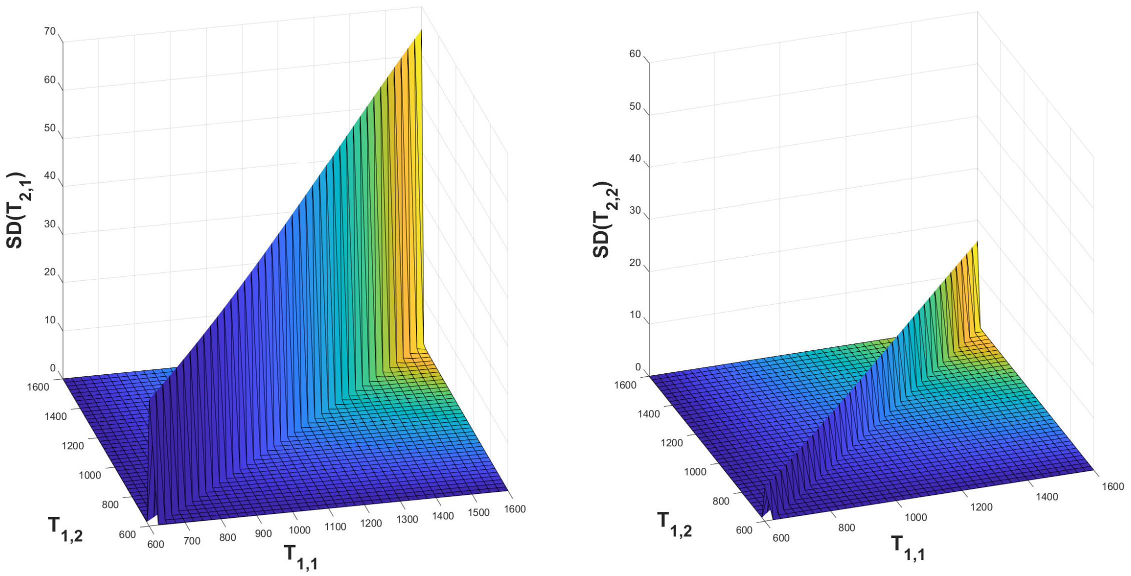

Figure 8. Results of the linearized analytical calculation of parameter SD's $SD(T_{2,1}), SD(T_{2,2})$ for a biexponential model, as a function of the $(T_{1,1}, T_{1,2})$.

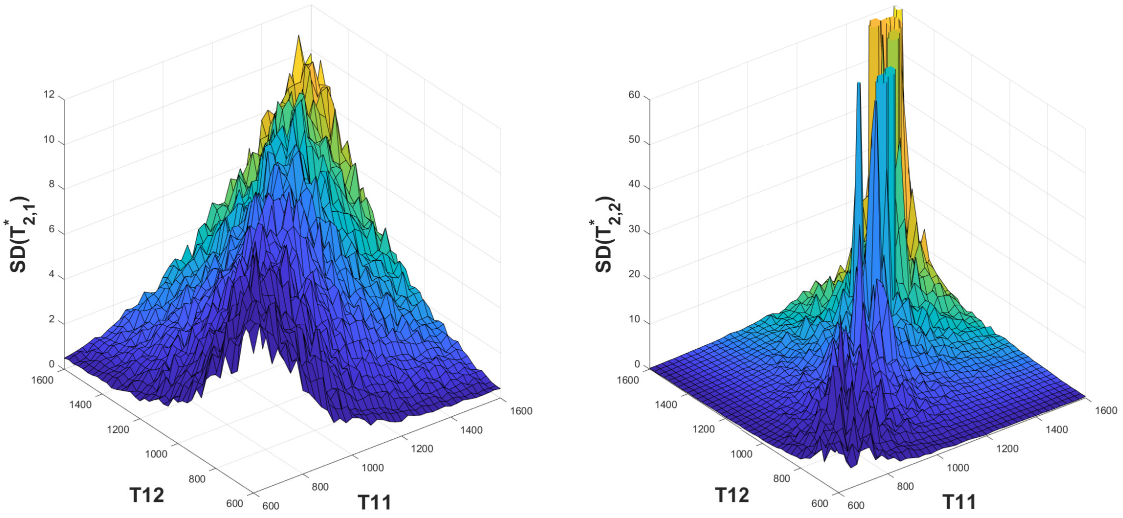

Figure 9. Results of the MC simulations of parameter SD's $SD\left(T^*_{2,1}\right), SD\left(T^*_{2,2}\right)$ for a biexponential model, as a function of the $(T_{1,1}, T_{1,2})$.

References

1. Trefethen, L. N. & Bau, D. *Numerical linear algebra* (Society for Industrial and Applied Mathematics, 1997).

2. Spencer, R. G. & Bi, C. A tutorial introduction to inverse problems in magnetic resonance. *NMR Biomed.* DOI: 10.1002/nbm.4315 (2020). E4315.
